# Post-Migration Education Among Refugees in the Netherlands

**DOI:** 10.3389/fsoc.2021.787009

**Published:** 2022-01-13

**Authors:** Frank van Tubergen

**Affiliations:** ^1^ Department of Sociology, Utrecht University, Utrecht, Netherlands; ^2^ Netherlands Interdisciplinary Demographic Institute, KNAW/University of Groningen, The Hague, Netherlands

**Keywords:** refugees, post-migration education, schooling, human capital, Netherlands

## Abstract

Refugees face significant barriers in the labor markets of western countries due to limited transferability of educational credentials. Post-migration education can increase refugees’ chances in the labor market, but little is known about the prevalence and underlying patterns of such post-secondary educational investments. I contribute to the literature by analyzing survey data from the Netherlands on post-migration education among more than 3,000 adult refugees who come from Afghanistan, Iran, Iraq, former Yugoslavia, and Somalia. I find that refugees’ investments in schooling depend on both pre- and post-migration characteristics. Results show that post-migration schooling is more common among adult refugees who are higher educated, who arrived at a younger age, who have applied for recognition of their foreign education, and who have (successfully) participated in integration and/or language courses. When refugees are kept in an asylum center for a longer time, they are less likely to invest in post-migration education.

## Introduction

In the past decades, the size of the refugee population in Europe has increased considerably. Studies show that refugees face significant barriers in the labor market. After the first 2 years of arrival, refugees’ employment rates are below 20–25% in EU countries (Dumont et al., 2016). Over time, refugees improve their position, but remain at a significant employment disadvantage compared to other migrants (Bakker et al., 2017; Brell et al., 2020). Research suggests that refugees’ vulnerable employment position is due to multiple factors (De Vroome and Van Tubergen, 2010; Hainmueller et al., 2016). A key barrier refugees face is the lack of returns to their educational credentials obtained in the country of origin (De Vroome and Van Tubergen, 2010; Tibajev and Hellgren, 2019; Damelang et al., 2020). Studies show that education acquired in the receiving country is associated with better labor market outcomes for refugees and other migrants (Kanas and Van Tubergen, 2010; Bakker et al., 2017; Lancee and Bol, 2017).

This study examines post-migration investments in education among refugees who arrived in the host country as adults (i.e., 18 years or older). Given the key role host-country schooling plays for refugees in their labor market integration, it is important to understand the conditions that promote and inhibit educational investments among adult refugees. I contribute to previous work in this field in two ways.

First, I look at refugees whereas the literature has focused on post-migration investments among family and labor migrants (Chiswick and Miller, 1994; Khan, 1997; Van Tubergen and Van de Werfhorst, 2007; Banerjee and Verma, 2012; Adamuti-Trache et al., 2013; Calvo and Sarkisian, 2015; Adamuti-Trache, 2016). An exception to this is the study of Damelang and Kosyakova (2021), who used a choice experiment to study educational preferences among refugees in Germany. I supplement their work by studying actual decisions rather than hypothetical preferences for investments. Refugees differ from family and labor migrant groups in various ways, such as in their settlement and integration process. Upon arrival, asylum seekers must apply for asylum, and during the application process they must stay in an asylum center. The impact of these refugee-specific conditions on educational investments has not been studied before.

Second, I examine in more detail the role of schooling obtained in the country of origin. Whereas earlier work on post-migration investments in schooling among immigrants has studied the impact of the level of education in the origin country or years of schooling (“vertical stratification”), I also study the effect of field of study (“horizontal stratification”). Little is known so far whether post-migration investments in education differ between fields of pre-migration education (e.g., medicine, economics, agriculture). Furthermore, I examine the impact of seeking recognition of foreign education. Earlier work shows that labor market outcomes for immigrants improve in case they have their foreign qualifications formally recognized (Damelang et al., 2020), but little is known about the impact of recognition of foreign education on post-migration investments in education.

The aim of this study is to assess the impact of pre-migration education (i.e., level and field) and various post-migration characteristics on refugees’ post-migration investments in schooling. I draw on a large-scale survey conducted in the Netherlands among more than 3,000 refugees from Afghanistan, Iran, Iraq, Somalia, and former Yugoslavia. I focus on the group of refugees who arrived in the Netherlands between age 18 and 64.

## Theory and Hypotheses

### Background

In the literature on post-migration investments in education, scholars have mainly used the Immigrant Human Capital Investment (IHCI) model, developed by Duleep and Regets (1999), to derive hypotheses about post-migration investments in education (Van Tubergen and Van de Werfhorst, 2007; Banerjee and Verma, 2012; Adamuti-Trache et al., 2013; Calvo and Sarkisian, 2015; Adamuti-Trache, 2016; Damelang and Kosyakova, 2021). According to this model, the decision to invest in post-migration schooling depends on the costs and benefits of these investments. Only when the benefits outweigh the costs, immigrants invest in post-migration education.

The cost-benefit calculation is argued to be a function of 1) settlement intentions, 2) skill transferability, and 3) opportunity costs. When immigrants intend to stay in the host country, investing in host-country education is more attractive, because the period in which one could use the newly obtained education is longer. Skill transferability matters too. The more strongly migrants are confronted with lack of returns to their origin-country qualifications, the more strongly their incentives to invest in host-country education. But opportunity costs play a role as well because investing in education means that earnings are forgone while studying. To organize the discussion of the hypotheses, I distinguish between the role of 1) pre-migration education, and 2) post-migration characteristics.

Before developing the hypotheses, it is important to situate the study in the context of the Netherlands. The Central Agency for the Reception of Asylum Seekers (COA) is responsible for the reception, support, and guidance of asylum seekers in the Netherlands. COA provides asylum seekers with accommodation and meals. During the asylum procedure, an asylum seeker receives a maximum of approximately €59 per week for food, clothing, and pocket money for other expenses. In the Netherlands, adult asylum seekers cannot follow education during the asylum procedure, and without residence permit they are also restricted in their right to work -although work restrictions have gradually relaxed in the past decades. If asylum seekers work, they have to pay COA for housing and other costs of living. In 2021 asylum seekers were not allowed to work for more than 24 weeks per year. They can keep 25% of the income for themselves, with a maximum of 185 euro per month.

When refugees in the Netherlands have been granted a residence permit, they can enroll in education. There are no tuition fees in the Netherlands for primary and secondary education, whereas fees for post-secondary education have remained below approximately 2000 euro per year. In addition, since 1986, Dutch students who were enrolled in post-secondary education received a grant of around 200–300 euro per month, to accommodate their study and cover costs for housing and living expenses. This study funding, however, has gradually been stripped down. In 1993, the study grant was converted into a loan and students only got the loan back if they obtained sufficient grades or passed their education. Since 2010, students no longer receive a study grant, but students can borrow money from the government on favorable terms to support themselves.

### Pre-Migration Education

The IHCI model suggests that post-migration investments in education become more rewarding when immigrants’ origin-country skills and credentials are undervalued compared to skills acquired in the destination country. Research shows that among non-western immigrants, including refugees, the returns to origin-country education in European countries are significantly lower than the returns to host-country schooling. The de-valuation of foreign educational credentials has been observed in Sweden (Tibajev and Hellgren, 2019), Italy (Fellini et al., 2018), Belgium (Kanas and Van Tubergen, 2014), the Netherlands (Hartog and Zorlu, 2009; De Vroome and Van Tubergen, 2010; Kanas and Van Tubergen, 2010), and in a cross-national study of several European countries (Lancee and Bol, 2017). In their study on Sweden, Tibajev and Hellgren (2019) find that official recognition of foreign credentials is associated with 4.4 percentage points higher probability of being employed, and 13.9 log points higher wage for those with employment. Also in Germany, it has been found that recognition of foreign education has a positive effect on employment (Damelang et al., 2020).

Given that origin-county education is undervalued, refugees -like other immigrants-have an economic incentive to invest in post-migration schooling (Friedberg, 2000). But such investments are also costly, i.e., there are “direct” costs (i.e., costs of getting an education), and costs of forgone earnings (opportunity costs). What matters most for refugees in the Netherlands are opportunity costs (i.e., forgone costs while not working), because public education is largely free in the Netherlands. In the IHCI model, it is argued that highly-educated immigrants invest more in post-migration schooling than lower-educated immigrants, because it is assumed that low skill transferability reduces the opportunity costs more than it reduces productivity. Higher-educated immigrants face higher opportunity costs, but they are also more efficient in obtaining additional schooling. Hence, I hypothesize that *highly-educated refugees invest more in post-migration schooling than lower-educated refugees* (H1).

Previous work has mainly looked at pre-migration level of education as a determinant of post-migration education, while little is known about the role of field of education. In the stratification literature, however, it has been shown that there are large differences in income and occupational status across fields of education (Gerber and Cheung, 2008) Consequently, it could be argued that for some fields of education, it is more rewarding to acquire additional diplomas. A medical specialist, for example, might strongly benefit from getting an official certificate in the host country, given the high earnings profile and the inability to work as a specialist without such a formal diploma. Someone trained in personal care and social services, however, may possibly receive higher returns to the origin-country education, and be able to acquire additional skills on the job. Because it is difficult to specify precisely for each field of education the expected costs and benefits of post-migration investments in schooling, I explore variations across fields of education in investments rather than formulating a testable hypothesis. The results can stimulate theory development, and hypotheses about the role of field of education can subsequently be tested in follow-up work.

### Post-Migration Characteristics

While young refugees automatically enroll in the Dutch educational system, the situation is different for refugees who arrived as adults. For them, after having obtained a residence permit, it may take time to get to know and understand the institutions in their new host country. The school system in the Netherlands is also quite complicated, because of its stratified, tracked system (Van de Werfhorst and Van Tubergen, 2007). Hence, finding the appropriate educational track, applying for enrollment, getting started with the education program, and obtaining a degree may take considerable time. A longer stay in the host country means that they have had more opportunities for educational investments. For this reason, I hypothesize that *the longer refugees stay in the host country, the higher their investments in post-migration schooling* (H2).

The opportunities for investing in education are hindered when refugees stay longer in an asylum center. In the Netherlands, as in other European countries, asylum seekers who apply for formal refugee status spend months or even several years in asylum centers. During their application process they cannot study, and have limited opportunities to work, as mentioned, but also interactions with ethnic majority members are restricted. Moreover, the uncertainty about the outcomes of the procedure may deter refugees from investing in host-country education (Kosyakova and Brenzel, 2020; Damelang and Kosyakova, 2021). Studies show that longer stay in an asylum center is negatively associated with employment in the Netherlands (De Vroome and Van Tubergen, 2010; Bakker et al., 2014), Switzerland (Hainmueller et al., 2016) and Germany (Kosyakova and Brenzel, 2020). Previous work also indicates that waiting time has negative health outcomes (Laban et al., 2008; Hvidtfeldt et al., 2020), which may lower refugees’ incentives and efficiency in post-migration educational investments. Because of these arguments and findings, I expect to see that *a longer stay in a reception center negatively affects refugees’ investments in post-migration schooling* (H3).

When asylum seekers have acquired a formal refugee status, they can decide to enroll in “integration” and language courses (De Vroome and Van Tubergen, 2010). In the Netherlands, integration courses aim to help refugees and other immigrants to become familiar with the Dutch society, i.e., its core institutions, norms, and values. I argue that participating in integration and language courses positively impact post-migration educational investments, for two reasons. First, by acquiring Dutch and learning about the Dutch society, it becomes easier for refugees to find their way to appropriate educational programs in the Netherlands. Because most programs are taught in Dutch, enrolling also becomes easier for those who have acquired the Dutch language. Secondly, participating in integration and language courses also signals a commitment to stay in the Netherlands. When refugees intend to settle in the Netherlands, it becomes more attractive to invest in education. For these reasons I expect to see that *enrollment in integration and language courses positively affects refugees’ investments in post-migration schooling* (H4).

Refugees, like other immigrants, can seek formal recognition of their foreign qualifications. In the Netherlands, the application process for diploma recognition is free. The impact of having received such recognition on post-migration investments is ambiguous, however, and I empirically test two hypotheses that go in opposite directions.

On the one hand are findings from previous research, which show that labor market outcomes for immigrants improve in case they have their foreign qualifications formally recognized (Damelang et al., 2020). This can be due to legal barriers to employment being removed (Damelang and Kosyakova, 2021), but also because recognition improves the signaling value of foreign credentials (Damelang et al., 2020). Either way, when refugees have their foreign education recognized, their opportunities in the labour market increase, which makes it less attractive to invest in education. For these reasons, one would expect to see that *refugees with recognized origin-country education are less likely to invest in post-migration schooling than refugees without recognized origin-country education* (H5a).

On the other hand, however, one could argue in the opposite direction, for two reasons. First, getting foreign educational credentials officially approved, opens opportunities for enrolling in educational programs. For example, to enroll in university in the Netherlands, one needs to have completed the pre-university track in secondary education (in Dutch: VWO), or to have obtained the propaedeutic year in higher vocational education (in Dutch: HBO). Only in case refugees have an equivalent foreign diploma to one of these two diplomas, they can enroll in Dutch university programs. Hence, foreign recognition of education opens up opportunities for pursuing education in the Netherlands. Second, refugees who seek recognition for their foreign qualification may also be a selective group, who are strongly committed to stay in the Netherlands. It takes time and effort to seek recognition, and thus those who do, might be more motivated than those who don’t. Thus, based on the idea that formal recognition of foreign credentials creates new opportunities for educational investments and also reveal commitment to stay in the host country, one could hypothesize that refugees with *recognized origin-country education are more likely to invest in post-migration schooling than refugees without recognized origin-country education* (H5b).

## Data and Measurement

### Data

Data are from the SPVA (Social Position and use of Provisions by Ethnic Minorities), which is a large-scale survey conducted in 2003 (ISEO, 2003; Schothorst, 2004). The survey was done among refugees from Afghanistan, Iran, Iraq, Somalia, and the former Yugoslavia. Respondents were randomly selected from municipal records in twelve larger cities in the Netherlands. These cities were chosen because of the concentration of these groups in larger cities, to reduce survey costs. The respondents were interviewed face-to-face in Dutch, English, or French. Respondents could choose in which of these three languages they would do the interview.

Response rates of the groups were between 43 percent (former Yugoslavians) and 55 percent (Afghans). The sources of non-response showed a similar pattern across the refugee groups, the most important being that about 25 percent of the people refused cooperation, about 15 percent of the people could not be contacted at the time of data collection, and about 5 percent could not participate because of language difficulties. Other sources of non-response include respondents for whom the address was incorrect and respondents who were in very bad health or had passed away. Analysis of the data shows that the distribution of the sample across age and gender strongly resembles that of the respective refugee population in the Netherlands (Schothorst, 2004).

Interviews were conducted with computer assisted personal interviewing among household members. A full questionnaire was presented to the so-called “heads of household,” which were often male. Partners and children older then 12 years were also interviewed. In this study, I combine the information from the head of the household and the partner. However, not all the variables that are included in this study were present in the shorter questionnaire that was used to gather information on the partners. Consequently, information on the time spent in reception centers is unavailable for the partner.

### Measurement

In the construction of the dependent variable, I follow previous work on post-migration investments among adult immigrants in the Netherlands (Van Tubergen and Van de Werfhorst, 2007). The main analysis is based on the dependent variable *schooling*, which has the outcomes 0) no schooling in the Netherlands, 1) uncompleted schooling (i.e., without diploma), and 2) completed schooling. This variable allows for several comparisons in terms of educational investments. First, I compare refugees with no schooling in the Netherlands 0) with those who obtained any schooling (1 or 2) -the latter group showing higher levels of post-migration investments in education. It is this contrast that is most in line with the hypotheses, and statistically significant differences regarding this contrast (in the right direction) are then regarded as confirmations for these hypotheses.

However, there might be considerable heterogeneity within the group of refugees who followed any schooling in the Netherlands: some refugees have been in school for only one or 2 months, whereas others may have successfully obtained education after years of study. Therefore, I also consider whether refugees completed their education. Although educational success in the Netherlands depends on many factors, it also captures refugees’ effort and willingness to invest in education. Therefore, I compare, within the group who obtained any post-migration education, those who were unsuccessful in getting a Dutch diploma 1) with those who completed their education 2).

Because the variable schooling does not say anything about the level of education followed, I additionally study *educational level* among refugees who have had received at least some schooling in the Netherlands. The highest educational level they followed in the Netherlands (with or without diploma) is measured in four categories 0) primary education, 1) lower vocational education (in Dutch: VMBO-kader, LBO, LTS), 2) secondary education, vocational education (MAVO, VMBO-TL, MBO, HAVO, VWO), 3) higher vocational training and university (HBO, WO). These additional, explorative, analyses supplement the main analysis rather than that they provide evidence to test the hypotheses.

For the variable *pre-migration level of education*, I use a measure indicating the highest level of education the respondent has followed in the country of origin. The measure includes six categories ranging from 0) no education at all, 1) less than 5 years of primary education, 2) more than 5 years of primary education, 3) lower secondary education, 4) higher secondary education, 5) to tertiary education. In additional analysis, I present results from *pre-migration years of schooling*. Questions about *pre-migration field of study* were asked to only those respondents who followed at least obtained secondary education in their country of origin. Answer categories were: 1) general; 2) languages, art, philosophy; 3) agriculture; 4) technical, science; 5) economics, administrative, law; 6) medical, 7) sociocultural; 8) personal and social care; 9) teacher; 10) other.

*Length of stay* in the Netherlands is measured as a continuous variable. To estimate the effect of *time spent in asylum centers*, I combined the time spent in three different types of reception facilities for refugees in the Netherlands: an “application center,” a “reception center,” and ⁄ or an “asylum center.”

Respondents were asked if they had ever attended an *integration course*, and if they had completed it with a diploma. I include four dummy variables, distinguishing between: 1) those who have never attended such a course, 2) those who have completed an integration course with a diploma, 3) those who did attend an integration course but have not received a diploma, and 4) those who are currently enrolled in an integration course. Regarding *language course*, I distinguish between those who have ever participated in such a course 1) and those who have not 0). Respondents were not asked if they had completed the course successfully.

*Recognition of foreign education* was asked in two steps. Respondents were asked if they had applied for formal recognition in the Netherlands, and if they had, what the outcome of the procedure was. I differentiate between 1) those who have not applied, 2) those whose application was pending at the moment of the survey, 3) those whose education was not recognized, or valued at a lower level, 4) those whose education was recognized.

Following earlier work on post-migration investments, I include several demographic factors as control variables. First, I include *national-origin groups*, as previous studies observed differences across origin groups in post-migration schooling (Khan, 1997; Van Tubergen and Van de Werfhorst, 2007; Calvo and Sarkisian, 2015). Second, I explore *gender* differences, as some research indicates that immigrant men more often invest in post-migration schooling than immigrant women (Chiswick and Miller, 1994), although this pattern has not always been found (Calvo and Sarkisian, 2015; Damelang and Kosyakova, 2021). Third, I include *age at arrival* in the Netherlands. Earlier studies showed that age at arrival was negatively correlated with post-migration investments, which may be due to reduced incentives to invest in education, shorter time horizon to stay in the host country, and reduced efficiency of learning (Van Tubergen and Van de Werfhorst, 2007; Damelang and Kosyakova, 2021). I include categorical variables for *age at migration* (18–19, 20–21, 22–23, 24–25, 26–27, 28–29, 30–39, 40–49, 50–65) because of possible nonlinear effects (Van Tubergen and Van de Werfhorst, 2007).

The original dataset includes a total of 3,547 refugees who are heads of household and 1,227 partners. I selected only first-generation refugees who arrived in the Netherlands between the ages of 18 and 65. I deleted respondents with missing data on one or more variables and excluded refugees who arrived before 1990 in the Netherlands -to minimize biases resulting from selective remigration, and institutional changes. After these selections, there are 2,563 heads of households and 826 partners. The analyses on the impact of waiting time in asylum centers is based on the sample that includes only heads of household. Table 1 provides an overview of the descriptive statistics of the independent variables included in the analyses.

**TABLE 1:** Descriptive statistics of independent variables.

	*N*	Mean (or proportion)	Sd	min	Max
Education in origin country					
None	3,389	0.102		0	1
Primary, < 5 years	3,389	0.022		0	1
Primary, > 5 years	3,389	0.185		0	1
Secondary, lower	3,389	0.138		0	1
Secondary, higher	3,389	0.316		0	1
Tertiary	3,389	0.237		0	1
Years of schooling in origin country	3,389	11.088	5.244	0	33
Field of education in origin country					
General	2,338	0.211		0	1
Language, art, philosophy	2,338	0.057		0	1
Agriculture	2,338	0.021		0	1
Technical, science	2,338	0.284		0	1
Economics, administrative, law	2,338	0.175		0	1
Medical	2,338	0.100		0	1
Sociocultural	2,338	0.031		0	1
Personal and social care	2,338	0.028		0	1
Teacher	2,338	0.051		0	1
Other	2,338	0.042		0	1
Length of stay in the Netherlands	3,389	8.527	2.950	1	14
Length of stay in asylum center*	2,563	0.902	1.274	0	10.5
Integration course					
Never followed	3,389	0.430		0	1
Followed, no diploma	3,389	0.143		0	1
Followed, currently enrolled	3,385	0.101		0	1
Followed, diploma	3,385	0.326		0	1
Participated in language course	3,389	0.452		0	1
Recognition of foreign education					
Never applied	3,389	0.770		0	1
Applied, currently pending	3,389	0.020		0	1
Applied, not recognized	3,389	0.118		0	1
Applied, recognized	3,389	0.090		0	1
National origin group					
Afghanistan	3,389	0.239		0	1
Iraq	3,389	0.242		0	1
Iran	3,389	0.199		0	1
Former Yugoslavia	3,389	0.182		0	1
Somalia	3,389	0.137		0	1
Female	3,389	0.427		0	1
Age at migration	3,389	29.70	8.950	18	64

Note: * = only asked among heads of households.

## Results

Table 2 presents descriptive findings for post-migration investments among adult refugees who arrived between 1990 and 2003 in the Netherlands. Around 27% of them participated in the Dutch school system. In a study of labor and family migrants in the Netherlands, coming from Turkey, Morocco, Suriname, and the Dutch Antilles, it was found that about 20% had received some schooling after migration (Van Tubergen and Van de Werfhorst, 2007). That study also reported that among those who obtained some schooling in the Netherlands around 47% was successful in completing their education. Among refugees, I find that, among those who followed any schooling in the Netherlands (12/27 = ) 56% received a diploma in the Netherlands. Taken together, these results provide some evidence to suggest that refugees invest more in post-migration education than family and labor migrants. Possibly, this is due to their forced reason of migration, which make refugees less-well prepared for the labor market (Damelang and Kosyakova, 2021).

**TABLE 2:** Post-migration investments in education among adult refugees in the Netherlands.

	*N*	%
Education participation in the Netherlands		
No schooling	2,460	73
Uncompleted schooling	423	12
Completed schooling	506	15
Total	3,389	100
Highest level of schooling followed in the Netherlands^a^		
Primary	104	11
Lower vocational	184	20
Secondary and higher vocational	350	38
Tertiary vocational college and university	273	30
Total	911	100

a Among refugees who have followed schooling in the Netherlands.

The main analysis is based on a multinomial regression model of three post-migration investments outcomes: no schooling, schooling but no diploma, and schooling with diploma (Table 3).

**TABLE 3:** Multinomial logistic regression of post-migration educational investments.

	P(Y = 1)		P(Y = 2)		P(Y = 3)	
	No schooling		Schooling, no diploma		Schooling, diploma	
Variables	dy/dx	se	dy/dx	se	dy/dx	se
Education in origin country (ref = secondary, lower)						
None	0.058*	(0.033)	0.031	(0.030)	−0.089***	(0.023)
Primary, < 5 years	0.128**	(0.054)	−0.024	(0.044)	−0.104**	(0.042)
Primary, > 5 years	0.026	(0.027)	−0.014	(0.021)	−0.012	(0.022)
Secondary, higher	−0.051**	(0.023)	0.006	(0.019)	0.045**	(0.019)
Tertiary	−0.124***	(0.027)	0.026	(0.022)	0.098***	(0.023)
Length of stay in Netherlands	−0.012***	(0.003)	−0.007***	(0.002)	0.019***	(0.002)
Integration course (ref = never followed)						
Followed, no diploma	0.038	(0.026)	0.017	(0.023)	−0.056***	(0.020)
Followed, currently enrolled	0.084***	(0.029)	0.030	(0.026)	−0.114***	(0.020)
Followed, diploma	−0.107***	(0.020)	0.046***	(0.017)	0.061***	(0.016)
Participated in language course	−0.076***	(0.018)	0.041***	(0.014)	0.035**	(0.015)
Recognition of foreign education (ref = never applied)						
Applied, currently pending	0.071	(0.048)	−0.033	(0.034)	−0.038	(0.042)
Applied, not recognized	−0.146***	(0.026)	0.091***	(0.023)	0.055***	(0.018)
Applied, recognized	−0.147***	(0.028)	0.101***	(0.026)	0.046**	(0.020)
National origin group (ref = Afghanistan)						
Iraq	0.019	(0.021)	−0.041***	(0.014)	0.023	(0.019)
Iran	−0.102***	(0.025)	0.013	(0.018)	0.089***	(0.022)
Former Yugoslavia	0.023	(0.023)	−0.052***	(0.015)	0.029	(0.021)
Somalia	0.063**	(0.025)	−0.025	(0.018)	−0.038*	(0.021)
Female	0.052***	(0.015)	−0.010	(0.012)	−0.043***	(0.012)
Age at migration (ref = 26–27)						
18–19	−0.176***	(0.037)	0.050	(0.031)	0.126***	(0.034)
20–21	−0.125***	(0.033)	0.069**	(0.027)	0.056**	(0.027)
22–23	−0.099***	(0.031)	0.040	(0.025)	0.059**	(0.026)
24–25	−0.059*	(0.031)	0.061**	(0.026)	−0.002	(0.025)
28–29	0.065**	(0.029)	−0.029	(0.022)	−0.036	(0.023)
30–39	0.062**	(0.024)	−0.016	(0.019)	−0.046**	(0.019)
40–49	0.118***	(0.028)	−0.044**	(0.021)	−0.074***	(0.023)
50–65	0.220***	(0.032)	−0.088***	(0.024)	−0.131***	(0.025)

Note: Presented are average marginal effects. ***p < 0.01, **p < 0.05, *p < 0.1. *N* = 3,385.

I find that pre-migration education is statistically significantly and strongly related to post-migration investments in schooling. Level of pre-migration education is positively associated with higher probabilities of getting any schooling in the Netherlands, and also with receiving a diploma. Each additional year of schooling in the country of origin lowers the probability of receiving no education in the Netherlands with 0.01, while it increases the probability of getting a diploma in the Netherlands with 0.01. When using years of pre-migration schooling instead of ordered categories of pre-migration educational level, I find the same pattern (Supplementary Table S1). These findings confirm H1.

Length of stay is positively associated with getting any schooling in the Netherlands. Over time, the probability to having received no schooling at all or having followed schooling without successful completion declines, whereas more and more refugees obtain a diploma. For each additional year of stay in the Netherlands, the probability of getting a diploma increases with 0.02, when controlling for the post-migration characteristics included in Table 3. These findings strongly confirm H2.

It was hypothesized that investments in integration and language courses would be positively associated with investments in post-migration schooling. I find that refugees who have ever participated in a Dutch language course are more likely to invest in any schooling in the Netherlands, increasing the probability of schooling with or without diploma about equally. Regarding integration courses, the data allow for more differentiation. It appears that, among those who ever followed an integration course, only those who have completed it with a diploma are significantly more likely to invest in schooling in the Netherlands. Refugees who were enrolled in an integration course at the moment of the survey had less often obtained a diploma than refugees who never attended an integration course, which could indicate that enrollment in such courses temporarily restricts refugees from schooling, but in the long run such courses, when completed successfully, may help them in obtaining education. Overall, these findings are in line with H4.

Regarding recognition of foreign education, two opposite predictions were formulated. I find that refugees who applied for recognition of their origin education (except for those whose application is pending) are more likely to invest in education in the Netherlands than those who never applied. This speaks against H5a, which predicted a negative link between foreign recognition and school investments, based on the idea that recognition would remove the legal barriers to employment. In this regard, the comparison between refugees who have applied and received recognition for their education with those who have applied but were unsuccessful is insightful. The results suggest that there is no difference between these two groups in their post-migration schooling investments, whereas the legal barriers are removed for one group and not the other. The empirics are more in favor of H5b, which hypothesized that foreign recognition increases the likelihood of post-migration investments in schooling. Possibly, it is not so much the case that recognition removes barriers to enter educational programs in the Netherlands, but rather that applying for foreign recognition reveals a stronger commitment and an intention to stay in the Netherlands.

With respect to demographics, I find evidence for differences between national origin groups, gender and age at migration. When taking pre- and post-migration characteristics into account, it appears that refugees from Iran are more likely to follow schooling in the Netherlands than other groups, whereas those from Somalia are the least likely to do so. Furthermore, I find that female refugees less often invest in schooling in the Netherlands than male refugees. Specifically, female refugees have four percent lower probability to get a Dutch diploma compared with males.

Age at migration has a strong impact on post-migration investments in schooling. Even among refugees who arrived as adults in the Netherlands, their age at arrival matters considerably. I don’t find that this effect has a strong cut-off point, and that beyond a certain age the age at arrival does not matter anymore. Instead, it appears that the age-at-arrival effect pertains across the entire range. For example, those who arrive at age 18 or 19 have a 0.176 lower probability to receive no schooling at all compared to those who were 26 or 27 at arrival, and their probability to get a diploma in the Netherlands is 0.126 higher. But also among higher age groups, i.e., 30–65, I find that those who arrive at a younger age invest more strongly in schooling.

To explore possible differences between fields of pre-migration education, I estimated a multinomial model among those who have obtained at least higher secondary education in the country of origin (for which information on pre-migration field of education is available). I find that refugees who have obtained a diploma in medicine in their country of origin are the most likely to invest in schooling in the Netherlands, whereas those who were trained as teachers invest the least (Table 4). The difference between these two fields of education is substantial (i.e., 0.14 higher probability of no schooling among refugees trained as teachers). Other than these two fields of education, however, differences appear small.

**TABLE 4:** Multinomial logistic regression of post-migration educational investments on field of education in origin country. Subsample of refugees who obtained at least secondary education in country of origin.

	P(Y = 1)		P(Y = 2)		P(Y = 3)	
	No schooling		Schooling, no diploma		Schooling, diploma	
Variables	dy/dx	se	dy/dx	se	dy/dx	se
Field of education in origin country (ref = technical, science)						
General	0.024	(0.028)	0.028	(0.022)	−0.052**	(0.024)
Language, art, philosophy	−0.009	(0.041)	0.004	(0.033)	0.005	(0.034)
Agriculture	0.040	(0.069)	−0.039	(0.062)	−0.002	(0.056)
Economics, administrative, law	0.028	(0.028)	−0.001	(0.023)	−0.026	(0.023)
Medical	−0.071**	(0.032)	0.040	(0.024)	0.031	(0.026)
Sociocultural	−0.033	(0.052)	0.053	(0.038)	−0.020	(0.046)
Personal and social care	−0.016	(0.062)	0.089**	(0.040)	−0.073	(0.062)
Teacher	0.070	(0.051)	−0.028	(0.042)	−0.042	(0.046)
Other	0.041	(0.051)	0.003	(0.040)	−0.045	(0.045)

Note: Presented are average marginal effects. Variables included in main analysis (Table 3) are included as control variables. Presented are only results for field of education in origin country. ***p < 0.01, **p < 0.05, *p < 0.1. *N* = 2,335.

I selected the subsample of heads of households to estimate the effect of length of stay in an asylum center, as this information was only asked among this group of respondents. Results are presented in Table 5. Findings show that the time refugees have spent in an asylum center statistically significantly increases the probability of no schooling in the Netherlands, even when controlling for length of stay in the Netherlands, having followed integration and language courses, and whether refugees have applied for recognition of their foreign credentials. The findings are in line with H3.

**TABLE 5:** Multinomial logistic regression of post-migration educational investments on length of stay in asylum center. Subsample head of households.

	P(Y = 1)		P(Y = 2)		P(Y = 3)	
	No schooling		Schooling, no diploma		Schooling, diploma	
Variables	dy/dx	se	dy/dx	se	dy/dx	se
Length of stay in asylum center	0.013**	(0.007)	−0.009*	(0.006)	-0.004	(0.006)

Note: Presented are average marginal effects. Variables included in main analysis (Table 3) are included as control variables. Presented are only results for length of stay in asylum center. ***p < 0.01, **p < 0.05, *p < 0.1. *N* = 2,563.

Thus far, results have been presented on refugees’ investments in schooling in the Netherlands. To elaborate on this, I explore among those refugees who have attended school in the Netherlands, the highest level of education they have followed. The results of the ordered logit model (with the same predictors as in Table 3) are presented in Table 6. Some of the predictors mirror the patterns regarding investments in education. Specifically, I find that refugees who follow higher levels of education in the Netherlands are those with higher levels of pre-migration education, and who have applied for recognition of foreign education.

**TABLE 6:** Ordered logit regression of highest educational level followed in the Netherlands. Subsample of refugees with schooling in the Netherlands.

Variables	Coef	Se
Education in origin country (ref = secondary, lower)		
None	0.111	(0.333)
Primary, < 5 years	−0.081	(0.824)
Primary, > 5 years	−0.095	(0.249)
Secondary, higher	0.603***	(0.202)
Tertiary	1.762***	(0.230)
Length of stay in Netherlands	−0.052**	(0.026)
Integration course (ref = never followed)		
Followed, no diploma	−0.645**	(0.285)
Followed, currently enrolled	−0.538	(0.333)
Followed, diploma	−0.409**	(0.191)
Participated in language course	−0.206	(0.184)
Recognition of foreign education (ref = never applied)		
Applied, currently pending	0.629	(0.552)
Applied, not recognized	0.956***	(0.180)
Applied, recognized	0.716***	(0.193)
National origin group (ref = Afghanistan)		
Iraq	0.019	(0.196)
Iran	0.633***	(0.199)
Former Yugoslavia	0.555**	(0.219)
Somalia	−0.069	(0.253)
Female	0.141	(0.138)
Age at migration (ref = 26–27)		
18–19	0.868***	(0.281)
20–21	0.262	(0.254)
22–23	0.229	(0.251)
24–25	0.133	(0.255)
28–29	0.001	(0.288)
30–39	−0.219	(0.227)
40–49	−0.537*	(0.318)
50–65	0.148	(0.778)
Thresholds		
First	−1.756***	(0.409)
Second	-0.334	(0.404)
Third	1.707***	(0.411)

Note: Presented are logits. ***p < 0.01, **p < 0.05, *p < 0.1. *N* = 911.

On the other hand, there are also some predictors that deviate from the patterns for schooling. To begin, age at migration does not reveal a “linear” trend; instead, I find that the youngest age group (i.e., 18–19) reaches substantially higher levels of education than the rest, but that beyond that age group, there is no significant difference. Length of stay in the Netherlands is negatively associated with educational level, whereas there is a positive link between length of stay and schooling. Also, it appears that having followed an integration course is negatively associated with educational level, and participating in a language course shows no relationship, whereas having followed integration and language courses is positively related with schooling.

## Conclusion and Discussion

In the past decades, the refugee population in Europe has strongly increased. Findings indicate that refugees face significant barriers in the labor market, and that many are unemployed or have jobs below their skill level (Bakker et al., 2017; Brell et al., 2020). A key factor that contributes to the disadvantaged position of refugees in the labor market, is the lack of returns to education obtained in their country of origin -a pattern that has been observed across European societies (De Vroome and Van Tubergen, 2010; Lancee and Bol, 2017; Tibajev and Hellgren, 2019; Damelang et al., 2020). Investments in post-migration schooling are important for the labor market incorporation of refugees, studies show (Bakker et al., 2017). However, to date, little research has been done so far on the factors that hamper and promote refugees’ post-migration investments in education. To shed more light on this important societal topic, this study used data on more than 3,000 refugees in the Netherlands.

The results of this study are largely in line with the Immigrant Human Capital Investment (IHCI) model developed by Duleep and Regets (1999). I find that post-migration schooling is a positive function of pre-migration level of education. Higher-educated refugees may be more efficient in getting additional education in the Netherlands, and also more motivated to overcome discounting of their foreign education. In addition, I find that it takes time for refugees to obtain schooling in the Netherlands, which may be due to the complexity of an unfamiliar education system and the time it takes to enroll in a new program. Results further show that age at migration negatively correlates with refugees’ post-migration investments. This is in line with the idea that older refugees have less incentives to obtain education, and also may be less efficient in getting additional schooling. Regarding each of these three factors, similar observations were made for family and labor migrants (Van Tubergen and Van de Werfhorst, 2007; Banerjee and Verma, 2012; Calvo & Sarkisian, 2015), thereby suggesting a pattern that is common among immigrants, regardless of migration motive.

However, in this study I go beyond these patterns that have previously been found among family and labor migrants and examine factors that have not been studied before in this field (i.e., the role of participating in language and integration courses, recognition of foreign education, pre-migration field of education), and also factors that are refugee-specific (i.e., time refugees spent in reception center).

Findings show that refugees’ (successful) participation in integration and/or language courses strongly correlates with post-migration schooling. This is in line with the idea that learning the host-country language, but also getting to understand the core institutions of the receiving society, is important for refugees in (successfully) completing schooling. Policies that facilitate access to such integration and language courses can therefore have “spillover” effects, as refugees (and possibly also family and labor migrants) not only acquire the host-country language, but also become better equipped to obtain schooling in the host country.

I find that refugees who applied for recognition of their foreign education are *more* likely to invest in schooling in the Netherlands. One possible explanation for this pattern is that when refugees get their foreign educational credentials officially approved, it opens opportunities for enrolling in educational programs. To test this underlying process, I differentiated within the group of refugees who applied for recognition between those who succeeded in getting their foreign education approved, and those who were not successful. I find that within this group, there is no difference between refugees who have and have not received formal recognition in their post-migration schooling. Hence, rather than supporting the idea that foreign recognition opens doors for following education, these findings suggest that the group of refugees who apply for recognition of foreign credentials might be a selective group of refugees, who are committed to stay in the host country, and therefore have more to gain from investments in schooling.

The results speak against the opposite hypothesis which was formulated about the role of foreign recognition. Findings provide no evidence to suggest that refugees who have received recognition for their foreign education have fewer incentives to obtain additional education. This prediction was based on the idea that formal recognition would remove legal barriers to employment, and thereby lower the incentives for investing in schooling. Possibly, even when having their foreign education officially approved, refugees still encounter significantly lower returns to the education they obtained in the home country. This is in line with studies showing that, although formal recognition of foreign higher education leads to improved employment outcomes, it does not completely remove the disadvantage of foreign credentials (Tibajev and Hellgren, 2019; Damelang et al., 2020).

Refugees’ post-migration investments in schooling also differ between fields of home-country education. Those who, in their sending country, obtained a diploma in medicine are the most likely to pursue education in the Netherlands, whereas I find that those educated as teachers invest the least. Possibly, such differences reflect heterogeneity in the returns and transferability of educational diplomas and skills. For example, refugees trained as doctors may not find a job due to the lack of portability of their human capital, and they may be strongly motivated to obtain additional education in the Netherlands because of the high earnings profile for doctors. The wages for teachers are significantly lower, and also the skills and competencies teachers have acquired may be more comparable across countries. Following-up on earlier work, which showed that the returns to foreign credential recognition varies across occupations (Damelang et al., 2020), more research is encouraged on the returns to fields of home-country education.

I also examined the role of a refugee-specific factor, which is the waiting time in the asylum center. Results show that when refugees are kept in an asylum center for a longer time, they are less likely to subsequently follow education in the Netherlands. Previous research found that waiting time reduces refugees’ employment prospects (De Vroome and Van Tubergen, 2010; Bakker et al., 2014; Hainmueller et al., 2016; Kosyakova and Brenzel, 2020), and that waiting time has negative health outcomes (Laban et al., 2008; Hvidtfeldt et al., 2020). This study adds to this growing literature that length of stay in asylum centers negatively impacts post-migration schooling. Possibly, this is because during their application process refugees have limited opportunities to work and study and also because the uncertainty about the outcomes of the procedure may deter refugees from investing in host-country education (Kosyakova and Brenzel, 2020; Damelang and Kosyakova, 2021). A recommendation for social policy is therefore to limit the length of the application procedure and the time asylum seekers stay in a reception center.

There are several limitations of this study which call for follow-up research. One is that this study was situated in the Netherlands, using cross-sectional data on refugee groups from the year 2003, which raises questions about the generalizability of findings to other periods and countries. Since 2003, study grants for higher education have become more restrictive in the Netherlands, possibly lowering refugees’ post-migration schooling. Also, investments in education may depend on conditions in the labor market. One argument is that favorable employment conditions raise the opportunity costs of education. In line with this idea, earlier research found that the unemployment rate in the country positively affected post-migration schooling among immigrants (Van Tubergen and Van de Werfhorst, 2007). Relatedly, one could argue that in countries with extensive social welfare programs, such as the Netherlands, Denmark, Norway and Sweden, the incentives to invest in post-migration schooling are lower than in countries with less-extensive welfare state services, such as the United States, United Kingdom, and Australia. To test these arguments, more research is needed on over-time and cross-country patterns of post-migration schooling of immigrants and refugees.

## Data Availability

The datasets presented in this study can be found in online repositories. The names of the repository/repositories and accession number(s) can be found below: https://doi.org/10.17026/dans-x7u-vkfm.
