# Supplementary material for: Post-Migration Education Among Refugees in the Netherlands
Source: Front Sociol. 2022 Jan 13;6:787009. doi: 10.3389/fsoc.2021.787009 (PMC8793622; doi:10.3389/fsoc.2021.787009)
Supplement: Supplementary file 1 [file Table1.DOCX]

**Supplementary material**

S1 Table. Multinomial logistic regression of post-migration educational investments on years of schooling in country of origin.

|  | P(Y=1)  No schooling | | P(Y=2)  Schooling, no diploma | | P(Y=3)  Schooling, diploma | |
| --- | --- | --- | --- | --- | --- | --- |
|  |  |  |  |  |  |  |
| Variables | dy/dx | se | dy/dx | se | dy/dx | se |
|  |  |  |  |  |  |  |
| Years of schooling in origin country | -0.010*** | (0.002) | 0.002 | (0.001) | 0.009*** | (0.002) |
|  |  |  |  |  |  |  |

Note: Presented are average marginal effects. Variables included in main analysis (Table 3) are included as control variables, except for dummy variables for education in country of origin. Presented are only results for years of schooling in origin country. *** p<0.01, ** p<0.05, *p<0.1. N = 3,385
